# Management of Children Admitted to Hospitals across Bangladesh with Suspected or Confirmed COVID-19 and the Implications for the Future: A Nationwide Cross-Sectional Study

**DOI:** 10.3390/antibiotics11010105

**Published:** 2022-01-14

**Authors:** Kona Chowdhury, Mainul Haque, Nadia Nusrat, Nihad Adnan, Salequl Islam, Afzalunnessa Binte Lutfor, Dilara Begum, Arif Rabbany, Enamul Karim, Abdul Malek, Nasim Jahan, Jesmine Akter, Sumala Ashraf, Mohammad Nazmul Hasan, Mahmuda Hassan, Najnin Akhter, Monika Mazumder, Nazmus Sihan, Nurun Naher, Shaheen Akter, Sifat Uz Zaman, Tanjina Chowdhury, Jebun Nesa, Susmita Biswas, Mohammod Didarul Islam, Al Mamun Hossain, Habibur Rahman, Palash Kumar Biswas, Mohammed Shaheen, Farah Chowdhury, Santosh Kumar, Amanj Kurdi, Zia Ul Mustafa, Natalie Schellack, Marshall Gowere, Johanna C. Meyer, Sylvia Opanga, Brian Godman

**Affiliations:** 1 Department of Paediatrics, Gonoshasthaya Samaj Vittik Medical College and Hospital, Savar, Dhaka 1344, Bangladesh; konachy56@yahoo.com; 2 Unit of Pharmacology, Faculty of Medicine and Defence Health, Universiti Pertahanan Nasional Malaysia (National Defence University of Malaysia), Kem Perdana Sungai Besi, Kuala Lumpur 57000, Malaysia; 3 Department of Paediatrics, Delta Medical College and Hospital, 26/2, Principal Abul Kashem Road, Mirpur-1, Dhaka 1216, Bangladesh; nusratnadia.nn@gmail.com; 4 Department of Microbiology, Jahangirnagar University, Savar, Dhaka 1342, Bangladesh; nihad@juniv.edu (N.A.); salequl@juniv.edu (S.I.); sifat.zaman9@gmail.com (S.U.Z.); 5 Department of Microbiology, Ad-Din Women’s Medical College, 2 Boro Mogbazar, Dhaka 1217, Bangladesh; lizablutfor@gmail.com; 6 Depatment of Paediatrics, Dhaka Medical College Hospital, 100 Ramna Central Shaheed Minar Area, Bakshi Bazar, Dhaka 1000, Bangladesh; dr.dilara123@gmail.com; 7 Department of Paediatrics, Mymensnigh Medical College Hospital, Dhaka-Mymensingh Road, Mymensingh Sadar, Mymensingh 2200, Bangladesh; arifdrmmc@gmail.com; 8 Department of Paediatrics, US-Bangla Medical College, Kornogop, Tarabo, Rupganj, Narayangonj 1460, Bangladesh; drenamul@gmail.com; 9 Department of Pediatrics, Green Life Medical College Hospital, Dhaka 1205, Bangladesh; malekabdul003@yahoo.com; 10 Department of Pediatrics, Asgar Ali Hospital, Distillary Road, Ganderia, Dhaka 1204, Bangladesh; njahan.jesy@gmail.com; 11 Department of Pediatrics, Bangladesh Specialized Hospital, Mirpur Road, Dhaka 1207, Bangladesh; Jamituj@gmail.com; 12 Department of Paediatrics, Holy Family Red Crescent Medical College Hospital, 1-Eskaton Garden Road, Dhaka 1000, Bangladesh; sumalaashraf@yahoo.com; 13 Department Paediatric Surgery, Cumilla Medical College Hospital, Cumilla 3500, Bangladesh; monty.bich@gmail.com; 14 Department of Paediatrics, Ad-din Women’s Medical College, 2 Boro Mogbazar, Dhaka 1217, Bangladesh; mahmudahasn@yahoo.com; 15 Department of Pediatrics, Cumilla Medical College Hospital, Cumilla 3500, Bangladesh; najninakhter114517@gmail.com (N.A.); dr.sihan@gmail.com (N.S.); 16 Department of Pediatrics, Rangpur Medical College, Rangpur 5400, Bangladesh; monikamz26@yahoo.com; 17 Department of Pediatrics, Evercare Hospital, Plot-81, Block-E, Bashundhara Residential Area, Dhaka 1229, Bangladesh; nahernurun004@gmail.com; 18 Department of Pediatrics, Enam Medical College and Hospital, Savar, Dhaka 1340, Bangladesh; shaheenssr7@gmail.com; 19 Department of Pediatrics, Sylhet M.A.G. Osmani Medical College Hospital, Medical College Road, Kajolshah, Sylhet 3100, Bangladesh; tanjina0407@gmail.com; 20 Department of Paediatrics, Center for Women and Child Health, Savar, Dhaka 1349, Bangladesh; drjebunnesa@yahoo.com; 21 Department of Paediatrics, Chattogram Medical College Hospital, Panchlaish, Chattogram 4203, Bangladesh; drsusmitabiswas@yahoo.com (S.B.); shaheen3388@gmail.com (M.S.); 22 Department of Paediatrics, Shaheed Syed Nazrul Islam Medical College, Kishorganj 2300, Bangladesh; dr.didar23@gmail.com; 23 Department of Paediatrics, Satkhira Medical College Hospital, Baka, Satkhira 9400, Bangladesh; mamunpedi71@gmail.com; 24 Department of Paediatrics, Meherpur District Hospital, Meherpur 7100, Bangladesh; drhabib1979@gmail.com; 25 Department of Paediatrics, Jashore Medical College Hospital, Jessore 7400, Bangladesh; bashiardanga@gmail.com; 26 Department of Paediatrics, Chattogram Ma Shishu Hospital Medical College, Chattogram 4100, Bangladesh; chyfarah@yahoo.com; 27 Department of Periodontology and Implantology, Karnavati University, Gandhinagar 382422, India; drsantoshkumar2004@gmail.com; 28 Strathclyde Institute of Pharmacy and Biomedical Sciences, University of Strathclyde, Glasgow G4 0RE, UK; amanj.baker@strath.ac.uk; 29 Department of Pharmacology, College of Pharmacy, Hawler Medical University, Erbil 44001, Iraq; 30 Center of Research and Strategic Studies, Lebanese French University, Erbil 44001, Iraq; 31 Department of Pharmacy Services, District Headquarter (DHQ) Hospital, Pakpattan 57400, Pakistan; zia.ucp@gmail.com; 32 Department of Pharmacology, Faculty of Health Sciences, University of Pretoria, Pretoria 0007, South Africa; natalie.schellack@up.ac.za (N.S.); u18064397@tuks.co.za (M.G.); 33 Division of Public Health Pharmacy and Management, School of Pharmacy, Sefako Makgatho Health Sciences University, Pretoria 0204, South Africa; hannelie.meyer@smu.ac.za; 34 Department of Pharmaceutics and Pharmacy Practice, School of Pharmacy, University of Nairobi, Nairobi 00202, Kenya; sopanga@uonbi.ac.ke; 35 Centre of Medical and Bio-Allied Health Sciences Research, Ajman University, Ajman P.O. Box 346, United Arab Emirates

**Keywords:** antibiotics, antimicrobial stewardship programs, Bangladesh, children, COVID-19, guidelines, hospitals, outcomes

## Abstract

There is an increasing focus on researching children admitted to hospital with new variants of COVID-19, combined with concerns with hyperinflammatory syndromes and the overuse of antimicrobials. Paediatric guidelines have been produced in Bangladesh to improve their care. Consequently, the objective is to document the management of children with COVID-19 among 24 hospitals in Bangladesh. Key outcome measures included the percentage prescribed different antimicrobials, adherence to paediatric guidelines and mortality rates using purposely developed report forms. The majority of 146 admitted children were aged 5 years or under (62.3%) and were boys (58.9%). Reasons for admission included fever, respiratory distress and coughing; 86.3% were prescribed antibiotics, typically parenterally, on the WHO ‘Watch’ list, and empirically (98.4%). There were no differences in antibiotic use whether hospitals followed paediatric guidance or not. There was no prescribing of antimalarials and limited prescribing of antivirals (5.5% of children) and antiparasitic medicines (0.7%). The majority of children (92.5%) made a full recovery. It was encouraging to see the low hospitalisation rates and limited use of antimalarials, antivirals and antiparasitic medicines. However, the high empiric use of antibiotics, alongside limited switching to oral formulations, is a concern that can be addressed by instigating the appropriate programmes.

## 1. Introduction

The principal focus on children in low- and middle-income countries (LMICs) during the COVID-19 pandemic has been to address key issues, including the poor uptake of vaccinations and the envisaged impact as well as behavioural issues as a consequence of lockdowns and social distancing measures [1,2,3,4,5,6]. This resulted in Bangladesh organising immunisation outreach services for children and increasing the number of home visits to address concerns [3,7]. This is because children have a lower risk of infection with COVID-19 than adults, with typically milder clinical manifestations [8,9,10,11,12,13,14] and an appreciable number are asymptomatic [11,15]. Cough, fever, diarrhoea, nausea and respiratory infections are generally the most frequent clinical characteristics of children with COVID-19, with boys typically more prone than girls [8,11,12,13,15,16]. Overall, it is estimated that approximately 6% to 10% of infected children experience severe disease, lower than rates seen in adults [11,12]. Consequently, the focus among children during the current pandemic has been on other infectious diseases and their prevention.

However, the focus on children is changing with new variants and their potential implications. In addition, whilst a lower percentage of children with severe symptoms are admitted to intensive care units (ICUs) in LMICs, deaths among the hospitalised children are higher [12].

There are also increasing concerns with the inappropriate use of antimicrobials across LMICs. Patients with COVID-19 in hospitals are typically administered antibiotics despite only a limited number having concomitant bacterial or fungal infections [17,18,19,20,21,22]. This includes hospitals in Bangladesh [23,24]. However, high inappropriate prescribing will increase antimicrobial resistance (AMR) rates, increasing morbidity, mortality and costs [25,26,27,28,29,30,31,32], with high rates of AMR already a concern in Bangladesh [33,34,35,36]. Children with COVID-19 can also develop Kawasaki Disease (KD)-like symptoms/hyperinflammatory syndromes [8,37,38,39] as well as experience leucopoenia with marked lymphopenia, hyponatremia, hypoalbuminemia, gastrointestinal and respiratory changes [37], potentially increasing ICU admissions.

There have also been concerns generally with the level of misinformation surrounding the management of COVID-19. This includes issues with hydroxychloroquine, lopinavir/ritonavir, remdesivir and ivermectin, with robust studies suggesting limited clinical benefit, although systematic reviews have suggested remdesivir can accelerate clinical improvement [40,41,42,43,44,45,46,47,48,49]. This is a concern as misinformation can increase morbidity, mortality and costs [50,51,52,53]. Concerns with misinformation have resulted in groups, including the World Health Organization (WHO) and the British Medical Journal (BMJ), providing evidence-based guidance [54,55,56,57,58]. International guidelines have also been developed to improve the management of children with COVID-19 in paediatric intensive care unit (PICU) countries [59]. The Ministry of Health and Family Welfare in Bangladesh also made national guidelines available from May 2020 onwards [60]. Similarly, the Bangladesh Paediatric Association has published its guidelines as this patient population is inherently a vulnerable one (Table 1) [61].

Consequently, we believe it is important to document the current management of children admitted to hospitals in Bangladesh with suspected or confirmed COVID-19, especially given concerns with antimicrobial use. In view of this, the objective of this study is to document current prevalence rates, treatments and outcomes of children with COVID-19, admitted to hospitals in Bangladesh in recent months. The findings can be used to suggest future quality improvement programmes in Bangladesh and other countries, if pertinent.

## 2. Results

We will first document the characteristics of patients admitted to hospitals across Bangladesh before documenting prescribed treatments and patient outcomes. One hundred and forty-six children were admitted with COVID-19 to the 24 hospitals taking part in this study, giving an overall prevalence of 3.7% (*n* = 3902, range = 0.55% to 29.5%) during the study period. COVID-19 was confirmed in the majority of admitted children by PCR tests (*n* = 111, 76%). The majority of admitted children were boys (*n* = 86, 58.9%) and were aged between 0 and 5 years (*n* = 91, 62.3%) (Table 2).

**Figure 1:**
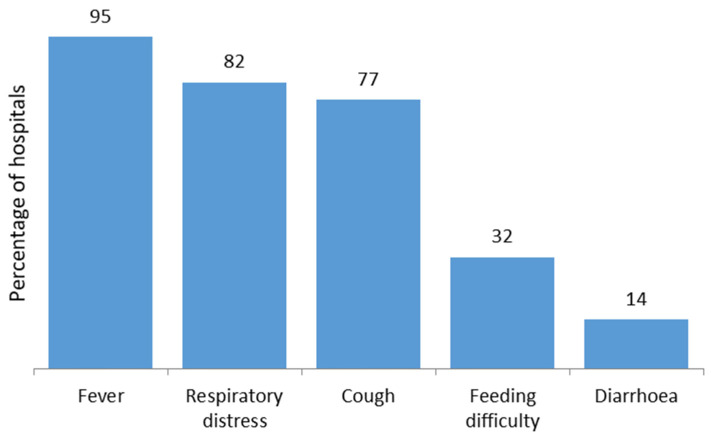
Reasons for hospital admission of children with COVID-19 (*n* = 146).

The consolidated principal reasons for hospital admission are documented in Table 3 based on pre-determined choices (Methodology Section 4.2), with only a limited number of children (*n* = 20, 13.7% including one referral) transferred to the PICU, typically with low oxygen saturation (Table 3, Figure 1). Children were typically admitted with more than one symptom, with Table 3 documenting the consolidated principal reasons for admission among participating hospitals. Potential reasons for admission to the PICU were also pre-determined and are documented in the Methodology Section 4.2. There were only a limited number of comorbidities among admitted children with COVID-19. These included bronchial asthma, dilated cardiomyopathy, congenital heart disease, dengue, nephrotic syndrome, malnutrition, pneumonia, sepsis and urinary tract infections.

Table 4 contains the consolidated list of antibiotics (class or specific) prescribed for children with suspected or confirmed COVID-19 among the participating hospitals. As seen, there was a considerable prescribing of antibiotics, with 86.3% (126/146) of children prescribed them, with almost all antibiotics administered empirically (98.4%—124/126). In general, antibiotics were prescribed from the WHO Watch list as opposed to the Reserve list. In addition, antibiotics were typically administered parenterally with generally limited prescribing of oral antibiotics, including switching, for between 3 and 14 days. Children were generally assessed after 2 to 3 days (Table 4).

There was no prescribing of hydroxychloroquine for any child admitted with COVID-19 in the surveyed hospitals and very limited prescribing of either antivirals, including remdesivir, or antiparasitic medicines, including ivermectin (Table 4). Steroids were prescribed to children in 13 of the surveyed hospitals (*n* = 13, 54.2%), with immune boosters, including vitamins C and D, prescribed to children in 14 of the participating hospitals (*n* = 14, 58.3% of hospitals).

There were lower rates of prescribing of antibiotics among those hospitals where the clinicians stated that they had followed the Bangladesh Paediatric Association guidelines (Table 1 and Table 5). However, there was no significant statistical association (*p* = 0.127) between the administration of antibiotics and the clinicians in the participating hospitals who had stated they had complied with the Paediatric Association guidelines when prescribing antibiotics to children with suspected or confirmed COVID-19 and those who had not (Table 5).

Encouragingly, at 1.4% (2/146), there were only a limited number of deaths among the children admitted with COVID-19, with 92.5% making a full recovery (135/146). The remainder still had complications during the study period.

## 3. Discussion

To the best of our knowledge, this is the first comprehensive study conducted among a range of both private and public hospitals across Bangladesh concerning the management of children hospitalised with suspected or confirmed COVID-19. Our findings build on the earlier study of Hussain et al. (2020), who found a higher mortality rate (13.3%) among children during the early stages of the pandemic [3], and the recent pilot studies conducted among children in Bangladesh and India [62,63]. The reduced mortality among children hospitalised with COVID-19 in our study (1.4%) compared with the study conducted by Hussain et al. (2020) may reflect improved clinical management as more knowledge is gained regarding the aetiology of COVID-19, especially with greater recognition of Kawasaki disease (KD)-like symptoms/hyperinflammatory syndromes among these children [8,37,38,39]. In addition, greater knowledge of treatment approaches has resulted in the development of updated guidelines (Table 1) as well as updated advice from groups such the WHO and the BMJ [54,58]. However, further research is needed on this topic before we can say anything with certainty. Having said this, only a low number of children were admitted to these general hospitals in Bangladesh with COVID-19 (Table 2), similar to the findings of other recent publications, including the recent pilot study in India [11,13,15,63].

Fever, cough and respiratory diseases were, again, the most frequent clinical characteristics of children admitted to the surveyed hospitals with COVID-19 (Table 3, Figure 1), with admission to hospital with suspected or confirmed COVID-19 also more common among boys (58.9%), similar to other studies [8,11,12,13,15,16,63].

Encouragingly there was limited prescribing of antiviral, antiparasitic and antimalarial medicines among the children with COVID-19 admitted to the surveyed hospitals (Table 4). This is in line with recommendations in the Bangladesh Paediatric guidelines (Table 1), with similar guidance in other countries [61,64,65]. This is important given concerns with the lack of effectiveness of these medicines in treating patients with COVID-19, despite the initial hype [40,41,43,44]. Encouragingly, the prescribing of immunomodulators such as vitamins and zinc also appeared to be in line with the paediatric guidelines (Table 1). However, we are beginning to see immunomodulators being discouraged in some LMICs [65].

However, there were concerns with the high empiric use of antibiotics among the children in the surveyed hospitals (Table 3), which is similar to their high use among adults admitted to hospitals with COVID-19 in Bangladesh [23,24]. These rates (86.3%) are higher than a recent pilot study undertaken in India, with rates of 69.4% [63]. This is despite, as mentioned, only a limited number of patients with COVID-19 across ages having concomitant bacterial or fungal infections [17,18,19,20,21,22,31,66]. In addition, the Paediatric Association guidelines advocate only the prudent use of antibiotics due to concerns with potential over-use (Table 1). However, the rates of antibiotic prescribing appear to be less among children where the clinicians have stated they followed paediatric guidelines (Table 5); however, this was not statistically significant. In any event, these high rates need to be addressed, going forward, to reduce future AMR.

Potential ways forward to improve the rational use of antibiotics among these children include instigating antimicrobial stewardship programmes (ASPs), combined with an additional COVID-19 module, and other educational activities; these currently do not exist. Such activities have been successful across LMICs to improve the rational use of antibiotics in a number of situations [28,29,32,36,67,68,69,70,71,72].

Future activities should also include encouraging antibiotic prescribing according to local antibiograms where initial prescribing is typically empiric, combined with instigating ASPs [35,73,74,75]. These combined activities should help reduce the extent of prescribing ‘Watch’ antibiotics in favour of the increased prescribing of ‘Access’ antibiotics, where pertinent (Table 2 and Table 4) [76,77]. ASPs could also potentially encourage earlier switching from parental to oral antibiotics where it is pertinent and practical to hasten earlier discharge and conserve resources [78,79,80]. This is important with the current practice of limited switching to oral antibiotics among the surveyed hospitals. We will be following up on these identified issues in future studies to improve antibiotic prescribing in Bangladesh, given continual concerns with high rates of AMR [33,34,35,81].

The seemingly low number of children admitted to these general hospitals in Bangladesh versus other conditions is important when considering future priorities with limited resources. This includes reviewing the clinical conditions of the other admitted children during this period without COVID-19 and whether resources should be focused on these children instead. In addition, there are key areas such as vaccinations to prevent future infections among children [28]. We are aware that in Bangladesh and other LMICs, vaccination rates have been appreciably compromised by lockdowns and other measures [1,2,3,4,5]. Whilst this was not a focus for this study, we are aware that the direct and indirect impact of COVID-19 on children has been underestimated. Children in LMICs are more likely to suffer from other comorbidities such as other respiratory tract infections, including pneumonia as well as malnutrition and HIV, which may complicate a COVID 19 diagnosis [82]. Consequently, we will also be following up on these issues as well in future research projects to provide additional guidance to all key stakeholder groups in Bangladesh and other countries.

We are aware of several limitations with this study. These included the fact that this was only a retrospective review of medical records without the ability to check with physicians regarding the rationale for any prescribing, especially of antibiotics. We were also limited by the extent of information that could be collected from the surveyed hospitals, and all cases of COVID-19 could not be confirmed by rt-PCR. We also could not analyse any differences between private and public hospitals as admission and other key criteria were not standardised. In addition, we extended the study period in each hospital in order to collect more data on the management of children with COVID-19. Consequently, our prevalence rates of admitted children could be overestimated. However, despite these limitations, we believe our findings are robust, given the number of participating hospitals, providing direction for future quality improvement programmes and other activities.

## 4. Materials and Methods

### 4.1. Study Setting and Design

The study was undertaken among a range of hospitals (*n* = 24) across Bangladesh, admitting children with suspected or confirmed COVID-19. Both private and public hospitals were included to ensure full representation, similar to point prevalence surveys conducted in other healthcare systems with a mixture of hospital types [83,84,85]. Initially, children in Bangladesh with COVID-19 were referred to public hospitals, mainly medical college hospitals. However, this changed, with private hospitals subsequently able to treat children admitted with COVID-19.

Private and public hospitals were not analysed separately due to anticipated different admission criteria, making comparisons problematic and difficult to justify. In addition, the aims of the study were to gain insights into current prevalence rates among children admitted with suspected COVID-19 to hospitals in Bangladesh, as well as insights into current management practices, rather than to assess differences in treatments and outcomes between different hospital types. This included the current prescribing of antimicrobials among hospitals in Bangladesh. The hospitals were purposely selected based on their ability to provide pertinent data as well as maximum variation in terms of their geography, location and size (Table 2).

This point prevalence survey was conducted based on standardised methodologies, with the data forms adapted to meet the specific requirements of the study and setting [62,83,84,85,86,87,88] (Supplementary Material File S1). Data were gathered from the medical records of children admitted to hospital with suspected or confirmed COVID-19 between July and November 2021. This included children subsequently admitted to PICUs, if such facilities existed in the hospital. Care in public hospitals in Bangladesh for these patients is typically provided without the need for co-payments. However, there are charges for patients admitted to private hospitals.

### 4.2. The Data Collection Tool and Analysis

A paper-based data collection tool was used by the investigators to collect the necessary information at the patient level. The specific forms were adapted from previous point prevalence surveys conducted in LMICs involving a number of co-authors [83,85,86,87,88,89,90,91,92]. This was combined with a synthesis of current guidelines for managing children with COVID-19 in Bangladesh (Table 1), a synthesis of the current literature [3,11,12,20,93] and a cognisance of the study aims and objectives. The data collection forms were pre-tested to ensure they would meet the study objectives. A pilot study was undertaken among a limited number of hospitals in Bangladesh [62] prior to full roll-out, with a similar pilot study undertaken in India [63]. The same forms were subsequently used in this multicentre study in Bangladesh as they were able to provide the relevant information to meet the aims and objectives of the study (Supplementary Material File S1).

There were no exclusion criteria. All children admitted on the day of data collection and during the previous nine days were included to give a combined total over a ten-day period.

The key data sets collected and the rationale for their inclusion in the data collection forms, where pertinent, are included in Table 6. The information was collected by the principal investigator in each hospital, with initial data collection taking place on one specific day, similar to other PPS studies. The medical records of patients admitted in the previous ten days were also viewed. Ten days were chosen for this study by the principal investigators because of the anticipated low numbers of children with COVID-19 actually admitted to general hospitals in Bangladesh on any specific day, with children with COVID-19 generally asymptomatic or with milder clinical manifestations versus adults [8,10,11,12,13]. Alongside this, there were anticipated low numbers of children with COVID-19 in the participating hospitals compared with other conditions of admitted children. In addition, as mentioned, the principal objective of the study was to gain knowledge of current management practices, including any concerns with antimicrobial use, rather than a robust assessment of prevalence rates. Children were also followed up, if necessary, to determine the average number of days they were prescribed antibiotics and when re-assessed.

Data were collated by the principal investigator in each participating hospital and subsequently entered into Microsoft Excel^®^ spreadsheets for analysis. As mentioned (Table 6), these contained drop-down menus with options for specific questions. The aggregated data for each hospital was subsequently transferred to the principal investigator collating the findings (BG) for analysis, with the ability to re-check and re-validate data when the need arose with the co-authors from the pertinent hospitals.

Descriptive statistics were typically used to summarise and collate the data using proportions (%) as appropriate, given the likely differences in admittance criteria between hospitals. However, the chi-square statistical test was used to assess whether there were significant differences in antibiotic prescribing between those hospitals that stipulated they followed current guidelines versus the remainder, with a *p*-value < 0.05 seen as significant (Table 5).

### 4.3. Patient and Hospital Anonymity and Ethical Approval

Patient anonymity was maintained throughout the study by the principal investigator in each hospital. Only aggregated anonymised data for each hospital was forwarded to the principal investigator collating the findings (BG) for analysis. Additionally, in the final analysis, hospitals were assigned a study identification number rather than each hospital being specifically identified (Hospitals 1 to 24).

Parents or guardians were not approached for consent since this was a retrospective study based on data collated from patients’ medical records, with no direct contact with children, their parents, or guardians. This is in line with previous point prevalence surveys undertaken by the co-authors [84,86,88,89,90,92,112].

The principal investigators orchestrated ethical approval for the hospitals and others involved in the study. The reference numbers are ibh/mirpur/2020/01; GSVMC/2021/346; BBEC, JU/M 2021/COVID-19/7 and AWMC/IRB-21 July 2021/028.

## 5. Conclusions

The low rates for the hospitalisation of children with COVID-19 among the surveyed hospitals in Bangladesh are encouraging and mirror the findings in other countries.

It was also encouraging to see limited prescribing of repurposed antimicrobials, including antimalarials, antivirals and antiparasitic medicines, given the hype that has surrounded these treatments, particularly hydroxychloroquine, which were without foundation once the findings from robust studies, including the WHO Solidarity and the UK Recovery studies, became known. This is important for promoting evidence-based approaches in the future.

However, high rates of the prescribing of antibiotics, often empiric, even among hospitals stating they are following the Paediatric Association guidelines, coupled with limited switching to oral formulations, are a concern. These issues need to be urgently addressed in Bangladesh, going forward, given the already high rates of AMR in the country coupled with ongoing resource pressures. This is important to reduce future morbidity, mortality, and costs. The instigation of local antibiograms and ASPs are key ways forward to improve future antibiotic use in hospitals in Bangladesh as well as LMICs generally. We will be monitoring such activities in the future.

## Figures and Tables

**Table 1:** Key recommendations for managing adults and children with COVID-19 in hospitals in Bangladesh (adapted from [60,61]).

**Adults/General (May 2020)**	**Treatment (General)** Moderate and severe cases should typically be treated in hospital, with more critical cases transferred to the ICU Monitoring of water and electrolyte balance as well as vital signs Oxygen saturation with oxygen therapy initiated if needed starting with low flow, including nasal catheter and mask oxygenation, before moving to high-flow oxygen therapy **Pharmacotherapy (moderate cases)** Steroids—methylprednisolone injection Early norepinephrine for hypotension Broad-spectrum antibiotics, e.g., meropenam IV/based on local antibiograms Remdesivir—discretion of consultant working in the hospital. If favipiravir has already been started in patients with moderate disease, this should be stopped in favour of remdesivir Consider tocilizumab and convalescent plasma therapy for cytokine storm/hemophagocytic lymphohistiocytosis **Severe disease** Additional oxygenation support for patients with severe disease If patients develop ARDS, intubation with mechanical ventilation will be needed; ECMO may be indicated in patients with refractory hypoxia in the ICU setting
**Children (November 2020)**	**Diagnosis and comorbidity** Ideally, PCR test to confirm the diagnosis and subsequent management on a designated COVID-19 ward or treatment area Comorbid conditions can include bronchial asthma, chronic kidney or liver disease and rheumatological conditions, including Kawasaki disease **General treatment and ICU** General treatment includes supportive care, electrolyte balancing and providing oxygen when necessary Children should be transferred to the paediatric ICU if they are experiencing severe/critical symptoms, respiratory failure requiring mechanical ventilation, shock, or organ failure. Treatment includes vasoactive drugs if required as well as balanced/buffered crystalloids **Recommended medicines for treating children with COVID-19** Antivirals—potentially reserved for children with severe acute respiratory syndrome. If remdesivir, then ideally part of ongoing clinical trials. Conflicting evidence regarding lopinavir/ritonavir Antibiotics—specific cases only, avoiding excessive use. 1st line—ampicillin plus gentamicin; 2nd line—ceftriaxone Corticosteroids—not used routinely. Low-dose dexamethasone may be beneficial in children with severe disease Vitamins—may be beneficial For suspected Kawasaki Disease/MIS-C—typically admit to paediatric ICU with supportive care, including antibiotics for suspected infections as well as steroids and other immune modifying therapies

ARDS—acute respiratory distress syndrome; ECMO—extracorporeal membrane oxygenation; ICU—Intensive Care Unit.

**Table 2:** Patient characteristics among the 24 participating hospitals during the study period.

Hospital	Date Survey Conducted	Total Number of Admitted Children during the Study Period	Total Number with COVID-19 (No.)	% of Admitted Children with COVID-19	Number of Children with Confirmed COVID-19 (No. and %)	Number of Boys (No.)	Number of Girls (No.)	0 to 5 Years of Age (no.)	6 to 10 Years of Age (No.)	11 to 18 Years of Age (No.)
1—Priv	20 July 2021	14	2	14.3%	2 (100%)	0	2	1	0	1
2—Priv	25 July 2021	18	1	5.6%	1 (100%)	1	0	0	1	0
3—Priv	30 July 2021	19	2	10.5%	1 (one susp.)	1	1	1	1	0
4—Priv	16 July 2021	32	2	6.3%	0 (2 susp.)	1	1	2	0	0
5—Priv	11 August 2021	40	5	12.5%	5 (100%)	2	3	2	2	1
6—Priv	30 July 2021	41	3	7.3%	3 (100%)	3	0	2	0	1
7—Pub	04 February 2021	44	13	29.5%	1 (12 susp.)	5	8	11	2	0
8—Priv	11 July 2021	50	5	10.0%	0 (5 susp.)	4	11	3	2	0
9—Priv	24 July 2021	61	5	8.2%	5 (100%)	3	2	1	2	2
10—Priv	11 August 2021	65	3	4.6%	3 (100%)	2	1	3	0	0
11—Priv	01 August 2021	68	6	8.8%	6 (100%)	5	1	3	3	0
12—Priv	30 July 21	75	15	20.0%	15 (100%)	9	6	4	9	2
13—Priv	10 August 2021	85	4	4.7%	4 (100%)	2	2	3	1	0
14—Priv	04 August 2021	103	14	13.6%	0 (14 susp.)	10	4	13	0	1
15—Pub	31 July 2021	105	1	1.0%	1 (100%)	1	0	1	0	0
16—Pub	31 August 2021	125	5	4.0%	5 (100%)	4	1	2	3	0
17—Pub	04 August 2021	68	3	4.4%	3 (100%)	2	1	2	0	1
18—Pub	31 July 2021	210	26	12.4%	26 (100%)	15	11	13	6	7
19—Pub	11 July 2021	256	3	1.2%	3 (100%)	2	1	2	1	0
20—Pub	08 October 2021	362	3	0.55%	3 (100%)	1	2	0	0	3
21—Pub	11 July 2021	382	3	0.8%	2 (1 susp)	2	1	3	0	0
22—Pub	08 November 2021	583	8	1.4%	8 (100%)	5	3	6	1	1
23—Pub	08 November 2021	442	5	1.1%	5 (100%)	1	4	4	1	0
24—Pub	17 July 2021	654	9	1.4%	9 (100%	5	4	9	0	0
		3902	146	3.7%	111—76% confirmed	86 (58.9%)	60 (41.1%)	91 (62.3%)	35 (24.0%)	20 (13.7%)

Column 3 includes children admitted during the study period and not on any specific day; Priv = private (including not for profit) hospital; Pub = public hospital; Confirmed—by PCR testing; susp = suspected.

**Table 3:** Rationale for admission to the hospital and those subsequently admitted to the PICU.

Hospital	Principal Documented Reasons for Hospital Admission for Children with Suspected COVID-19 during the Study Period	Total Number of Children Subsequently Admitted with COVID-19 to PICUs	Principal Reasons for PICU Admission
1—Priv	Respiratory distress	2	Low oxygen saturation, comorbidities
2—Priv	Fever, coughing, vomiting	0	Not applicable
3—Priv	Fever, cough, low SPO_2_, respiratory distress, vomiting, diarrhoea	0	Not applicable
4—Priv	Fever, cough, respiratory distress	0	Not applicable
5—Priv	Prolonged fever, respiratory distress, diarrhoea	1	Low oxygen saturation, extensive involvement in high-resolution CT scan
6—Priv	Prolonged fever, cough, respiratory distress	0	Not applicable
7—Pub	Fever, cough, respiratory distress	0 (1 referred) *	Unexplained bleeding
8—Priv	Prolonged fever, breathing difficulties, diarrhoea	1	Shock
9—Priv	Fever, cough, feeding difficulties	1	Shock, myocarditis
10—Priv	Respiratory distress, cough, feeding difficulties	0	Not applicable
11—Priv	Fever, cough, feeding difficulties	2	Low oxygen saturation, respiratory distress, shock
12—Priv	Fever, respiratory distress, feeding difficulty	0	Not applicable
13—Priv	Prolonged fever, cough, breathing difficulty/respiratory distress	0	Not applicable
14—Priv	Fever, respiratory distress, feeding difficulty	0	Not applicable
15—Pub	Fever, cough, respiratory distress	0	Not applicable
16—Pub	Prolonged fever, cough, Breathing difficulties/respiratory distress, diarrhoea	0	Not applicable
17—Pub	Fever, cough, respiratory distress	0	Not applicable
18—Pub	Fever, cough, low SPO2, respiratory distress, vomiting	9	Fever, shock, low oxygen saturation, vomiting, feeding difficulties
19—Pub	Fever, cough, respiratory distress	0	Not applicable
20—Pub	Fever, cough, respiratory distress	1	Low oxygen saturation and comorbidities
21—Pub	Prolonged fever, cough, respiratory distress	0	Not applicable
22—Pub	Fever, cough, respiratory distress	2	Perinatal asphyxia, feeding difficulty, low oxygen saturation
23—Pub	Fever, cough, respiratory distress	0	Not applicable
24—Pub	Prolonged fever, respiratory distress, feeding difficulties	0	Not appliable

PICU = Paediatric Intensive Care Unit; Priv = private hospital; Pub = public hospital; SPO_2_ = Saturation of peripheral oxygen; * referred to the PICU in another hospital.

**Table 4:** Clinical management of children with COVID-19 among the 24 hospitals.

Hospital	Number and % Prescribed Antibiotics	Empiric or Following CST	Principal Antibiotics Prescribed (Actual or Class) for Children with COVID-19	Antiviral Medicines Prescribed	Antiparasitic Medicines Prescribed	Duration of Antibiotic Prescribing (Days)	Clinical Assessment of Antibiotics (Days after Start of Treatment)
1—Priv	2 (100%)	All Empiric	Carbapenem, cephalosporins	No	No	4–7	3
2—Priv	1 (100%)	All Empiric	Ceftriaxone	No	No	5	5
3—Priv	2 (100%)	All Empiric	Ceftriaxone, ciprofloxacin	No	No	3 and 5	3–5
4—Priv	2 (100%)	All Empiric	Ampicillin, aminoglycosides, cephalosporins	No	No	5	2
5—Priv	5 (100%)	All Empiric	Cephalosporins, quinolones	Remdesivir (2 patients)	No	7	2
6—Priv	2 (66.7%)	All Empiric	Cephalosporins	No	No	5–7	5–7
7—Pub	13 (100%)	All Empiric	Ceftriaxone, amikacin, meropenem	No	No	5–7	3
8—Priv	5 (100%)	All Empiric	Carbapenem, aminoglycosides, cephalosporins	No	No	5–7	3
9—Priv	2 (40%)	All Empiric	Vancomycin, meropenem, co-amoxiclav	Remdesivir (1 patient)	No	7–10	3
10—Priv	3 (100%)	All Empiric	Ceftriaxone	No	No	10	3
11—Priv	4 (66.7%)	All Empiric	Ceftriaxone, ciprofloxacin, amikacin	Remdesivir (1 patient)	No	3–7	3
12—Priv	2 (13.3%)	1 Empiric/1 CST	Aminoglycosides, cephalosporins, quinolones	No	No	5–7	Not recorded
13—Priv	4 (100%)	All Empiric	Aminoglycosides, carbapenems, cephalosporins	No	Ivermectin (1 patient)	10	3–5
14—Priv	14 (100%)	All Empiric	Aminoglycosides, penicillin, cephalosporins	No	No	5–7	3
15—Pub	1 (100%)	All Empiric	Cefixime	No	No	7	3
16—Pub	5 (100%)	All Empiric	Aminoglycosides, carbapenems, cephalosporins	No	No	7–10	2–3
17—Pub	3 (100%)	All Empiric	Ceftriaxone, ceftazidim, gentamicin	No	No	3–7	3
18—Pub	26 (100%)	All Empiric	Ceftriaxone, ceftazidim, meropenem, vancomycin, flucloxacillin, amikacin	No	No	5–11	5–10
19—Pub	3 (100%)	All Empiric	Ceftriaxone, meropenem	No	No	7	3
20—Pub	3 (100%)	All Empiric	Penicillin, carbapenem, aminoglycosides	Remdesivir (3 patients), acyclovir (1 patient)	No	8–14	3
21—Pub	3 (100%)	All Empiric	Amikacin, meropenem, clarithromycine	No	No	7	2
22—Pub	7 (87.5%)	6 Empiric/1 CST	Ceftriaxone, amikacin, meropenem	No	No	4–7	3
23—Pub	5 (100%)	All Empiric	Ceftriaxone, amikacin, meropenem	No	No	7–14	3
24—Pub	9 (100%)	All Empiric	Aminoglycosides, carbapenem, macrolides	No	No	7 days (minimum)	3
Total	126 children—(86.3%)	124 empiric (98.4%)		8 children (5.5%)	1 child (0.7%)		

Antibiotics could also be prescribed for underlying comorbidities; Priv = private (including not for profit) hospital; Pub = public hospital.

**Table 5:** Prescribing of antibiotics and adherence to Bangladesh Paediatric Association guidelines.

Guideline adherence	Number of Children Administered Antibiotics	Number of Children Not Administered Antibiotics	% Administered Antibiotics
Hospitals where clinicians stated that they had followed the Paediatric Association guidelines	74	15	83.1% (*n* = 89)
Hospitals where clinicians stated that they had not followed the Paediatric Association guidelines	52	5	91.2% (*n* = 57)

Total number of children = 146.

**Table 6:** Key data sets collected and their rationale.

Key Data Sets	Rationale
Number of paediatric patients being treated in the hospital during the ten-day period and the number with COVID-19	To gain insight into current prevalence rates among children admitted to hospitals in Bangladesh compared with other conditions
Whether COVID-19 was suspected or confirmed—confirmed with PCR tests	To gain additional insight into current COVID-diagnostic practices
The ages of admitted children	Patients’ ages were broken down into 3 bands: 0–5 years; 6–10 years; 11–18 years for comparative purposes based on the pilot study in both Bangladesh and India [62,63]
Principal reason for admission to hospital with actual/suspected COVID-19	Potentially up to 3 principal reasons for admission could be recorded among participating hospitals These were taken from a drop-down menu in the Microsoft Excel^®^ spreadsheets and consisted of (i) breathing difficulties/respiratory distress; (ii) prolonged fever; (iii) cough; (iv) diarrhoea; and (v) feeding difficulty/vomiting These reasons were taken from the current literature, combined with input from paediatricians in Bangladesh, and tested in the pilot study [8,9,11,12,13,62] Consolidating the reasons would assist with analysis and comparisons with published studies
Comorbidities	Based on evidence amongst adults that certain comorbidities do have an impact on morbidity and mortality associated with COVID-19 [94,95]
Number of children admitted to PICU and the rationale	The potential reasons for admittance taken from the literature and input from paediatricians in Bangladesh included: (i) severe respiratory distress/low O_2_ saturation; (ii) shock; (iii) coagulation disorders/thromboembolic manifestations; and (iv) extensive lung involvement in high-resolution CT scans (HRCTs) [9,13,62] These potential reasons were standardised in the data collection forms for ease of recording and analysis and tested in the pilot study
Number of children prescribed antibiotics and the antibiotics prescribed (by ATC Level 4 Grouping or individual antibiotics), and whether empiric or following CST findings	This was assessed given concerns with potential over-prescribing coupled with guidance from the Bangladesh Paediatric Association, advocating prudence [61] Antibiotic prescribing also assessed against the WHO Access, Watch, or Reserve (AwaRe) list, given the increasing importance of this list to guide future antimicrobial policies and practices [76,86,88,96,97] The list of antibiotics prescribed for admitted children in specific hospitals was provided in a menu in the Case Report Forms (Supplementary Material File S1) and consolidated for tabulation and analysis
Route of administration, whether the rationale for antibiotic prescribing was re-assessed, and, if so, after how many days, and total length of antibiotic prescriptions	Given prior awareness that antibiotics administered to children admitted to hospital are usually administered by IV To determine whether: ○ There was any scaling down to oral antibiotics, as this can shorten hospital length of stay [91,98,99]. If so, the extent ○ There was any documented rationale for the antibiotics prescribed when re-assessed, especially with high empiric use, to help guide future quality improvement programmes among hospitals in Bangladesh
The extent of prescribing of antivirals, e.g., remdesivir, antimalarials, e.g., hydroxychloroquine, and antiparasitic medicines, e.g., ivermectin	Assessed, given concerns with their effectiveness and safety as more robust data became available, and should be reserved if administered (antivirals) according to the Bangladesh Paediatric Guidelines [40,43,44,46,48,61] Potentially, remdesivir for the management of patients in hospital with moderate to severe COVID-19 requiring oxygen, in the national Ministry of Health guidelines issued in Spring 2020 [60]—discretion of the hospital consultant
The extent of prescribing of dexamethasone and other steroids, including methylprednisolone	Seen as potentially beneficial, especially among hospitalised patients, and endorsed in the guidelines [46,61,100]
Use of supplements/immune boosters including vitamins C or D or zinc	Discussed in the Bangladesh Paediatric Guidelines, with publications suggesting potential benefit [61,101,102]
Adherence to current guidelines, including those developed by the Bangladesh Paediatric Association —Table 1 [50]	Adherence to robust guidelines is increasingly recognised as a key marker of quality used in the Global PPS studies as well as across hospitals in LMICs [83,103,104,105], given the concerns that can exist among LMICs [106,107,108] Published studies have shown that adherence to guidelines improves antimicrobial prescribing, as seen with the management of surgical site infections across LMICs as well as with the monitoring of prescribing guidance, built into antimicrobial stewardship programmes [28,67,70,109] However, adherence is not monitored and potentially enforced as the Bangladesh Paediatric Association is a non-government organisation. This is different to the situation that can exist across countries, sectors and disease areas [28,110,111]
Outcome—fully recovered, morbidity or mortality	Pertinent study endpoints Taken from the medical records of children during the study period by the principal investigator in each hospital
Possible costs (principally private hospitals) (based on local currency)	These were based on possible charges by the hospital (typically private hospitals) No attempt was made to undertake costing studies in public hospitals as this was not the objective of the paper

PICU = Pediatric Intensive Care Unit.

## Data Availability

Further data regarding the study is available upon reasonable request.

## Supplementary Materials

The following are available online at https://www.mdpi.com/article/10.3390/antibiotics11010105/s1, File S1: Bangladesh: COVID-19 Pediatric Case Management.
