# Supplementary material for: Management of Children Admitted to Hospitals across Bangladesh with Suspected or Confirmed COVID-19 and the Implications for the Future: A Nationwide Cross-Sectional Study
Source: Antibiotics (Basel). 2022 Jan 14;11(1):105. doi: 10.3390/antibiotics11010105 (PMC8772946; doi:10.3390/antibiotics11010105)
Supplement: Supplementary file 1 [file antibiotics-11-00105-s001.zip › antibiotics-1513724-supplementary.pdf]

Supplemantary Materials

File S1. Bangladesh: COVID-19 Pediatric Case Management

Pediatrician Name: Date of the survey:

Name of the hospital:

E-mail:

Orcid Id:

		**Admitted in Ward**	**Admitted in PICU**
Total patients in the children's ward including up to the previous 10 days			
Number and % of patients in the children's ward with COVID-19			
Primary reasons for admission of children with diagnosed/ suspected COVID-19 to the hospital (Tick/ Circle up to 3)		• Prolonged Fever.<br>• Breathing Difficulty/ Respiratory Distress.<br>• Cough.<br>• Diarrhea.<br>• Feeding Difficulty/Vomiting.<br>• Others- Specify	
Number of children admitted to PICU with covid-19 during the study period			
Primary reasons for admission to the PICU with diagnosed COVID-19 (Tick/ Circle up to 3)			• Severe respiratory distress/ Low oxygen saturation<br>• Shock<br>• Coagulation disorder/ Thromboembolic manifestation<br>• Extensive lung involvement in HRCT<br>• Co-morbidities
Age of patients with COVID-19	0-5 years		
	6-10 years		
	11-18 years		
Patients' Sex (Mention total number)	Male		
	Female		

Underlying conditions and co-morbidities (mention up to top 3 for admitted patients)			
Does Your Hospital Have a Specific Policy/ guideline to Manage pediatric patients based on the Pediatric Association Guidelines – Yes/ No			
If antibiotics prescribed, total number and % of patients receiving an antibiotic			
Antibiotics prescribed	Based on Culture Sensitivity Test (CST) findings (Number and %)		
	Empirically (Number and %)		
Name of Antimicrobials & Other prescribed Medicine + ATC code	Antibiotics (Tick/ Circle top 3)	Total number of children with COVID 19 prescribed antibiotics	
		Aminoglycoside	
		Carbapenems	
		Cephalosporins	
		Macrolide	
		Penicillin	
		Quinolones	
		Tetracycline	
		Others (please specify)	
	Antimalarial	Number of children with COVID 19 prescribed antimalarials	
		Hydroxychloroquine	
		Others	
	Antiviral (Number and % of patients)	Number of children with COVID 19 prescribed antivirals	
		Remdesivir	
		Others (please specify)	

	Antiparasitic (Number and % of patients)	Number of children with COVID 19 prescribed antiparasitics	
		Ivermectin	
		Others (please specify)	
	Anti-inflammatory (Number and % of patients)	Dexamethasone	
		Methylprednisolone	
		Toclizumab	
		Others (please specify)	
	Supplementary	Vit C	
		Vit D	
		Zinc	

Formulation of Antimicrobials	Oral (Number and %)	
	Parenteral (IV) (Number and %)	
Average duration of Prescribed Antimicrobials		
After prescribing antibiotics, clinically reassessed or not	Yes (After How Many days)	
	No	
Recovery rate during study period	Mortality (number and %)	
	Morbidity (Number and %)	
	Full recovery (Number and %)	
Duration of Hospital stay		
Total Expense (In Average Bangladeshi Taka – Private hospitals)		
	Private Hospital	

- **If any patient is referred to PICU of other hospital, please mention the cause of referral such as low oxygen saturation or others.**
